# EEG signatures of low back and knee joint pain during movement execution: a short report

**DOI:** 10.3389/fresc.2023.1216069

**Published:** 2023-08-16

**Authors:** Sabata Gervasio, Ali Asghar Zarei, Natalie Mrachacz-Kersting

**Affiliations:** ^1^ Neural Engineering and Neurophysiology Group, Department of Health Science and Technology, Aalborg University, Aalborg, Denmark; ^2^ REDO—Neurosystems, Aalborg, Denmark; ^3^ Institut of Sport and Sportscience, Albert-Ludwigs-Universität Freiburg, Freiburg, Germany

**Keywords:** musculoskeletal pain, knee pain, low back pain, electroencephalography, brain, movement, EEG

## Abstract

Chronic musculoskeletal pain has a high prevalence between European citizens, affecting their quality of life and their ability to work. The plastic changes associated with the occurrence of chronic musculoskeletal pain are still not fully understood. The current short report investigated the possible changes in brain activity caused by pain during movement in two of the most common musculoskeletal pain disorders in Denmark, knee pain and low back pain. Electroencephalography (EEG) was recorded from 20 participants (5 participants with knee pain, 5 with low back pain and 10 healthy controls). Participants with pain performed a movement that evoked pain in the area of interest, and the healthy controls performed the same movement. Electromyographic (EMG) signals were also collected to identify movement initiation. No differences were observed in brain activity of participants with pain and healthy controls during rest. During movement execution, though, participants with pain showed significantly higher event related synchronization in the alpha and beta bands compared to healthy controls. These changes could be related to higher cognitive processing, possibly due to the attempt of suppressing the pain. These results highlight the importance of assessing cortical activity during movement to reveal plastic changes due to musculoskeletal pain. This adds to our knowledge regarding plastic changes in cortical activity related to musculoskeletal pain in different locations. Such knowledge could help us identify neurophysiological markers for clinical changes and contribute to the development of new treatment approaches based on neuromodulation such as neurofeedback.

## 1. Introduction

Musculoskeletal pain refers to discomfort in muscles, ligaments, tendons and bones, usually following unusual activity or tissue inflammation, injury, overuse (1) or wears due to health transformation linked to aging (2). When it persists for longer than three months and after the expected time for tissue healing, the pain becomes chronic (3). Chronic musculoskeletal pain has a high prevalence, affecting around 100 million European citizen (4). Individuals with musculoskeletal pain experience symptoms like pain and fatigue, that affects their social life and financial capabilities as it likely limits their ability to work significantly (4). Musculoskeletal pain is therefore associated with notable costs for both the individual and society (5). While treatments such as physical therapy and exercise have been shown to be beneficial on function, amount of pain, and in general of the quality of life of individuals with pain (6), little attention has been paid to the understanding of chronic musculoskeletal pain. Chronic pain results from two processes: peripheral and central sensitization (2). These processes can result in neuroplastic change, which indicates changes in function, structure, and organization of the nervous system in response to internal factors, as from the afferent visceral system, and external factors, as from peripheral stimuli (2, 7). For instance, chronic inflammation in somatic structures could alter sensory afferents and lead to plastic changes in the nervous system (2).

Electroencephalography (EEG) is a noninvasive method that enables recording of electrical neuronal activity and therefore can contribute to our understanding of changes in brain function due to chronic pain (8). Individuals with chronic pain have been showing to have a distinct brain oscillatory signature, showing an increase in theta and alpha power at rest, compared to healthy controls (9, 10). Differences have also been observed between cortical activity in acute and chronic pain individuals, with beta power increasing and slower wave activity, such as delta, theta and alpha power, decreasing when the pain increases (11). However, changes in brain activity have mainly been investigated in neuropathic pain (10, 12), in induced pain or during rest. Research over the last years shows that musculoskeletal pain induces cortical changes (13–15). However, these studies are mainly conducted during rest, and provided non-consistent results (16–18). In our previous study we investigated the EEG signature of chronic elbow pain during a movement showing significant differences between participants with pain and healthy controls in alpha and beta band during the planning and execution of movements (19).

The aim of the present short report is to investigate possible changes in brain activity during movement in patients with chronic pain in the knee or lower back compared to healthy controls. Knee and low back pain were chosen as these are two of the most common musculoskeletal pain disorders in Denmark (20). Information regarding changes in cortical activity during movement related to pain in different pain locations could help identify neurophysiological markers for clinical changes, that may explain neurological changes in chronic pain and assist clinicians in making decision about treatment (21). Modulation of these neurophysiological markers could also be used as new pain treatment, for instance, through a neurofeedback approach (8, 11).

## 2. Methods

### 2.1. Participants

Ten volunteers with chronic musculoskeletal pain (seven females, 36 ± 11 years old) and ten healthy volunteers (seven females, 33 ± 12 years old) participated to the study. Of the ten participants with pain (PP), five experienced pain in the knee and in the lower back region. The PP and healthy controls were age and gender matched. Inclusion criteria for PP included the presence of lower back or knee pain for a minimum of three months and the occurrence of pain during movement. The volunteers did not suffer of any other neurologic or musculoskeletal disorders. None of the participants made use of medications. All participants were right-handed. Participants characteristics, as age, height and weight, pain location and duration for PP, are indicated in Tables 1, 2. In accordance with the Helsinki Declaration, all participants were informed about the experimental procedures and signed a consent before taking part to the study. The study was approved by the Local Ethics Committee (N-20140041).

**Table 1:** Pain participants characteristics.

Age (years)	Height (cm)	Weight (kg)	Pain location	Years of pain
23	174	65	Right knee	9
44	164	92	Right knee	11
50	158	72	Lower back	30
33	159	64	Right knee	31
51	166	75	Lower back	10
43	165	65	Lower back	10
28	174	70	Left knee	10
41	178	88	Lower back	1.5
23	169	62	Right knee	3
25	161	58	Lower back	6

**Table 2:** Healthy controls characteristics.

Age (years)	Height (cm)	Weight (kg)
28	167	72
24	181	68
28	162	60
59	165	60
27	168	51
49	178	64
26	168	85
29	178	75
26	172	59
28	168	58

### 2.2. Experimental protocol

The study was conducted in a quiet room. Participants were seated upright in a chair, with arms resting on their thighs, and their knees bent at approximately 90°. Participants were asked to perform a movement which provoked pain. The movement depended on the area affected by musculoskeletal pain. PP with pain in the knee, performed a knee extension. PP affected by pain in the lower back, performed an extension of the lower back. Healthy participants performed both knee extension and low back extension. The movement was identified and described to the participants and the participant performed three repetitions of the movement for familiarization before the data collection was initiated. The participants were asked to relax the area affected by musculoskeletal pain between movements, in order to avoid muscle contractions during resting. During data acquisition, the participants performed two rounds of 25 movements repetitions. The movements were self-paced, with a minimum of 10 s rest between movements. A self-paced movement instead of a cued movement was used as it has been shown that the expectation of the cue can affect brain activity, specifically alpha blocking appears before stimulus/cue onset (22). Moreover, self-paced movement are more representative of real-life situations. During the experiment, one experimenter observed the participants continuously to ensure the correct execution of the movement. Participants were provided with verbal feedback in case the movement deviated from the original movement or if the rest between the movements was shorter that 10 s. The two rounds were separated by 2 min of rest. Healthy controls followed the same protocol as participants with pain, but controls performed both movements, therefore a total of 50 knee extensions and 50 low back extensions. Healthy controls started randomly with knee or low back extensions. During data collection, Electromyographic (EMG) and Electroencephalographic (EEG) signals were recorded continuously. After every fifth repetition, the participant was asked to report the level of pain experienced during the movement using a Numeric Rating Scale (NRS). The NRS ranged from 0 to 10, where 0 indicated the absence of pain, and 10 indicated the worst imaginable pain.

### 2.3. Data acquisition

EMG was acquired to identify movement initiation. Disposable EMG electrodes (Neuroline 720 silver/silver-chloride, AMBU A/S, Denmark) were attached on the participants skin following appropriate skin preparation. The placement of the electrodes depended on the movement executed, and therefore the location of the pain. For PP with knee pain, the electrodes were placed on the m. vastus lateralis. For PP suffering from low back pain, the electrodes were placed on m. erector spinae. For the healthy controls, the electrodes were placed on both m. erector spinae and m. vastus lateralis, as controls performed both knee extensions and low back extensions. Electrodes were placed according to the SENIAM recommendations (23).

EEG signals were collected using a 64-channel EEG cap (g.GAMMA cap2, gTec, Austria) and a g.Hlamp-RESEARCH amplifier (gTec, GmbH, Austria). The EEG cap included active electrodes and was placed in accordance with the standard international 10–10 system with a ground in the AFz position and a reference electrode placed on the earlobe. All signals were collected using the g.HIamp amplifier, sampled at 1,200 Hz and stored for offline analysis. Signals were visually inspected to ensure a good signal to noise ratio before data acquisition was initiated. In case of poor signal quality, the experimenter attempted to reduce the impedance of the selected electrode by applying extra gel or ensuring this reached the scalp. The electrode impedance was kept below 30 kΩ throughout the data collection. To minimize eyes and head movements during data collection, the participants were instructed to fixate a cross sign on a wall placed approximately 2 m from the participants. The cross sign was maintained at the same height during all tasks of data collection.

### 2.4. Data processing

Data was exported and processed using MATLAB (version 2019b, The MathWorks, Natick, MA). EMG signals were filtered using a 6th order Butterworth, high pass filter with a cut-off frequency of 20 Hz to remove movement artifacts and was smoothed using a 6th order Butterworth, low-pass filter with a cut off frequency at 50 Hz (24). The onset of each movement was flagged using Teager–Kaiser Energy Operator (24) from the EMG data and visually reviewed a by an EMG expert. EEG data was re-referenced to the averaged reference. Based on the movement initiation identified with the EMG, the EEG data was segmented in time windows of 6 s duration, from 4 s before movement initiation to 2 s following movement initiation. This window was selected as we were interested in possible changes in brain activity during movement preparation and on movement initiation and in the resting time between movements. The independent component analysis (ICA) algorithm (FastICA) was employed to detect and extract eyeblink and muscle artifact components. Subsequently, the ADJUST algorithm was utilized to identify and eliminate the contaminated independent components (ICs) through an unsupervised approach, which was further validated manually. The total number of ICs estimated was equal to the number of channels which was 62 (ground and reference channels not included) (number of removed ICs: 10 ± 3). Next, trials with amplitudes exceeding ±100 µV were discarded due to suspected artifact contamination (total of 3 ± 2 trials). Finally, epochs were visually inspected by an EEG expert. The number of remaining epochs following pre-processing was 47.6 ± 1.03.

Time-frequency analysis was used to evaluate the brain activity between controls and participants suffering from musculoskeletal pain. As an indication of changes in power across time, event-related spectral perturbations (ERSPs) were extracted from the EEG data by using a wavelet transform and the average power across trials in each subject. The amplitude of the frequency components was extracted for each participant and for the time windows −4 to 2 s using a three-cycle Morlet-based wavelet transformer (Hanning-tapered window, frequency range from 3 to 45 Hz) in order to extract the ERSPs (18, 25). The formula used to calculate the ERSP was:

$$\mathrm{ERS}P_{\log}(\;f,t)=10\log_{10}\left(\frac{\frac{1}{n}\sum_{k=1}^{n}{\left|F_{k}(\;f,t)\right|}^{2}}{\mu_{B}(f)}\right)$$

Where n is the total number of trials, and $F_{k}(\;f,t)$ is the spectral estimate at frequency *f* and time point *t* for trial *k*. $\mu_{B}(f)$ is the mean spectral estimate for all baseline points at frequency *f*.

To evaluate the alteration in EEG activity on electrodes located close to the motor cortex as the possible differences between controls and PP during movement preparation (MP) and execution (ME), scalp maps based on the ERSP were calculated in the alpha (8–12 Hz) and beta (13–30 Hz) bands using the following time windows: MP1 (−400 to −200 ms), MP2 (−200 to 0 ms), ME1 (0–200 ms), ME2 (200–400 ms), ME3 (400–600 ms), ME4 (600–800 ms), and ME5, (800–1,000 ms). ERSP maps were extracted for each participant and time-frequency points were used for the statistical analysis.

### 2.5. Statistics

Data as NRS and age was screened for normality using a Shapiro–Wilk test. In case of not normally distributed data, an independent-samples Mann–Whitney *U*-test was applied to check for differences between control and participants with pain. Since the NRS was collected every 5 repetitions, thus 10 times in total for each participant, the average value of the NRS score for each participant was used for statistical analysis. ERSPs were assessed using a non-parametric test (26). Data is presented as mean ± standard deviation in case of normal distribution and median [interquartile range] in case of non-normally distribute data.

Channels with statically significant difference between the control and the PP were extracted for each time-frequency window. The significance level for multiple comparisons was corrected using False Discovery Rate (FDR). A one-way ANOVA based on non-parametric cluster-based permutation methods (26, 27) was performed on ERSP values across subjects in order to identify possible significant differences in time-frequency activity between healthy controls and participants with pain. Groups were considered as levels for the analysis. To identify time-frequency areas in which the groups showed significant differences in ERSP, non-parametric, cluster-based permutation was used. ERSP clusters were identified as adjacent time-frequency points with statistical differences between groups. Significance level was set at *p* ≤ 0.05.

## 3. Results

### 3.1. Participants and pain perception

No significant age difference was found between participants with pain and healthy volunteers (Mann–Whitney *U* = 53, *n*1 = *n*2 = 10, *p* = 0.85 two-tailed) confirming that participants were correctly age matched. The median and interquartile range for the NRS score were 5.883 (4.00) for pain and 0 (0) for the healthy participants. Participants with pain had a significantly higher pain rating compared to healthy controls (Mann–Whitney *U* = 100, *n*1 = *n*2 = 10, *p* < 0.01 two-tailed). As expected, only participants with pain reported pain (NRS values higher than 0) during movement execution.

### 3.2. Event related spectral perturbation in knee pain participants

**Figure 1:**
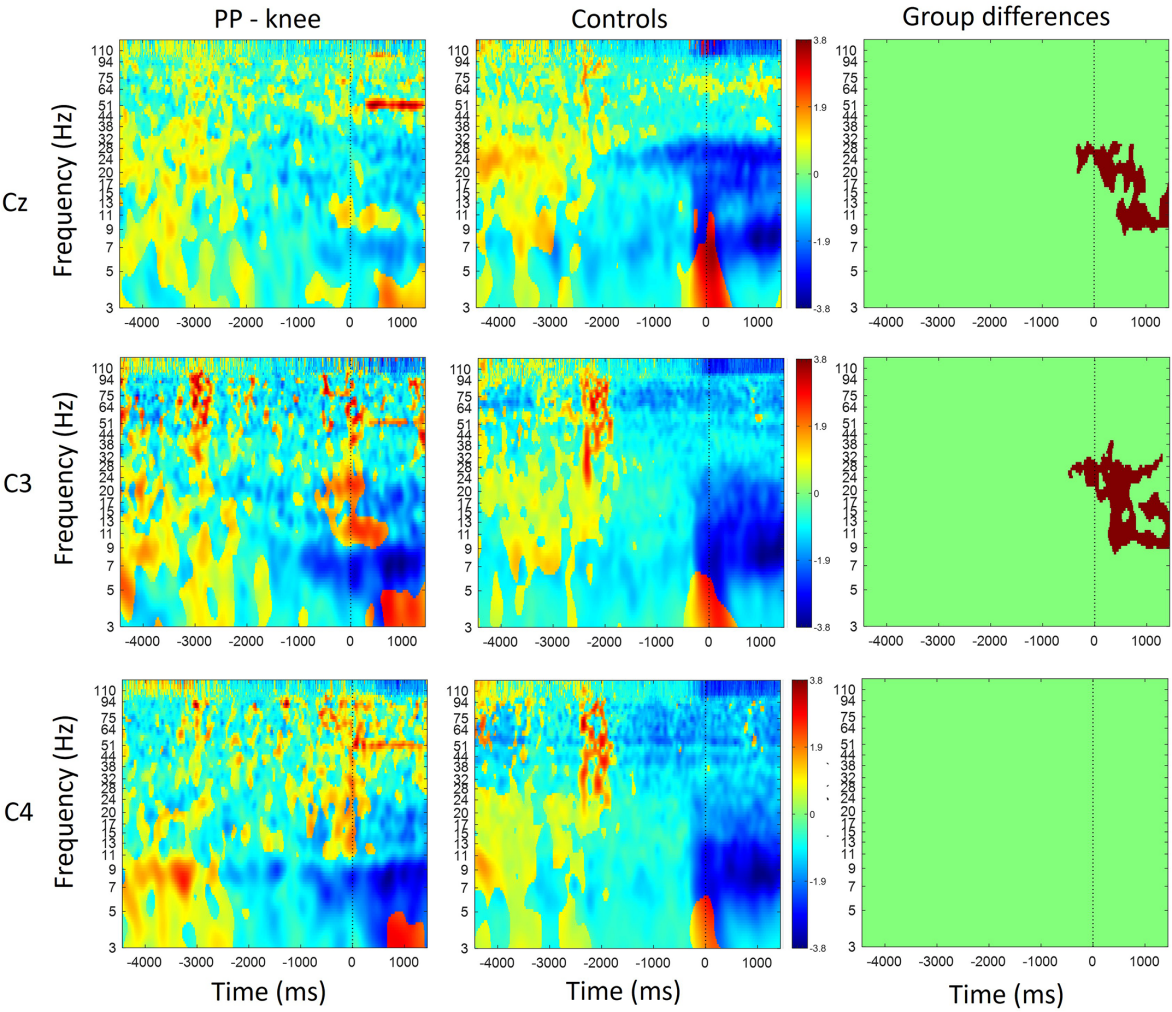
Time-frequency maps for controls and participants with knee pain over the Cz, C3 and C4. Movement onset is indicated by the vertical line (time 0). Regions of statistical differences (*p* < 0.05) are reported on the right column. Statistical differences were observed in channel Cz and C3 from around movement initiation and during the movement execution indicating an increase in oscillatory activity in the alpha and beta band in the participants with pain compared to healthy controls. No differences were observed in the resting phase between movements, that is until 2 s prior to movement initiation.

Figure 1 shows the time-frequency maps for controls and patients with knee pain for Cz, C3 and C4. An increase in oscillatory activity was observed around and following the movement initiation in the patient group compared to the control group. Regions of statistical differences (*p* < 0.05) were observed in channel Cz and C3 from around movement initiation and during the movement execution (Figure 1, right column). The region of statistical difference indicates an increase in power in the alpha and beta bands. No differences were observed during the resting phase that is until 2 s prior to movement initiation.

**Figure 2:**
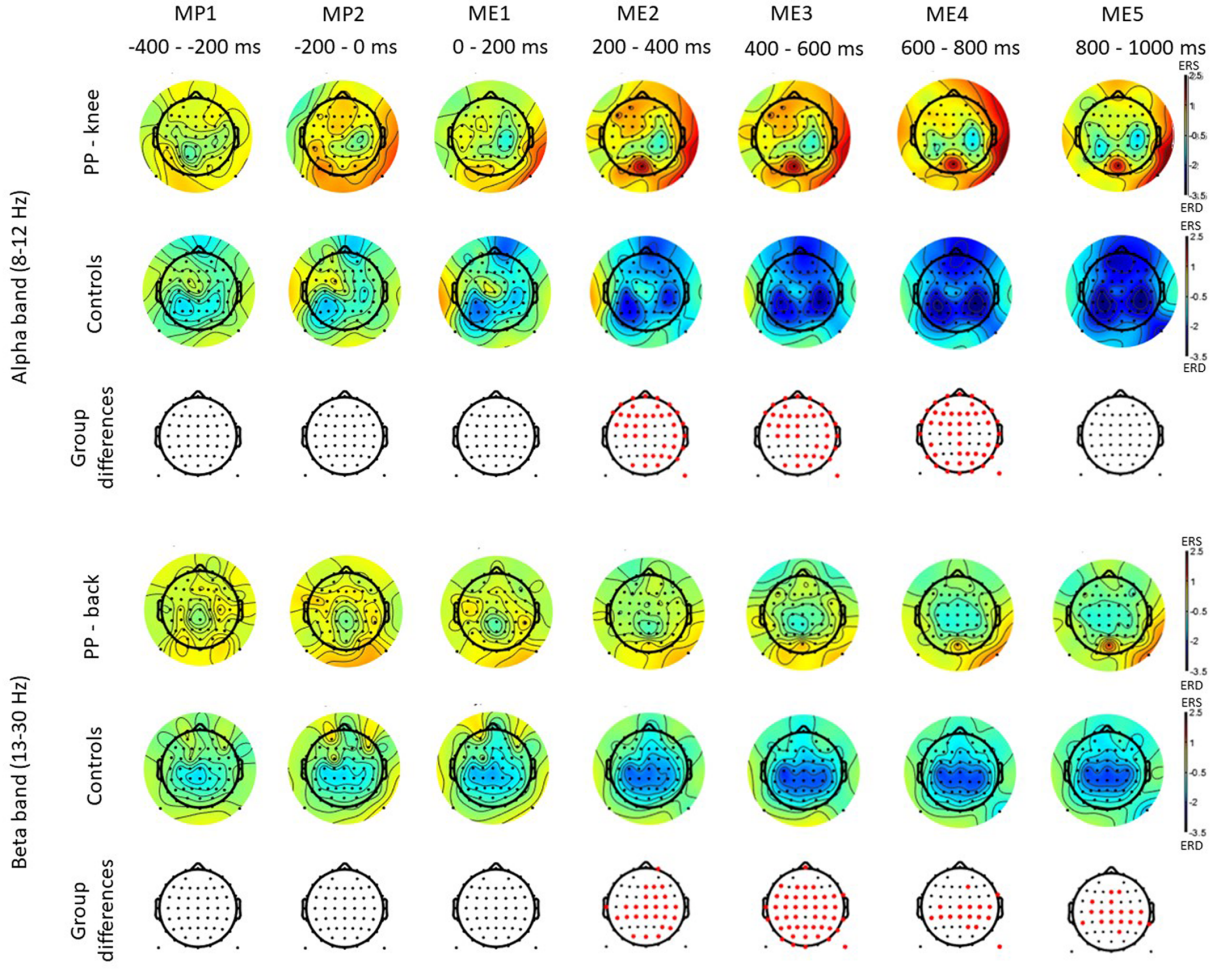
Scalp maps for the alpha and beta bands in time windows of 200 ms duration related to movement preparation (MP) and execution (ME) in participants with pain (PP) in the knee joint and healthy controls. The channels with statistical differences (*p* < 0.05) between controls and participants with pain are indicated in the lower row with red dots. Statistical differences between groups indicate an increase in alpha power between 200 and 800 ms (ME) following movement initiation and an increase in beta power between 200 and 1,000 ms (ME) following movement initiation in participants with pain compared to healthy controls.

Figure 2 shows scalp maps for the alpha and beta bands in time windows of 200 ms duration related to movement preparation and execution. In the figure, the channels with statistical differences (*p* < 0.05) between controls and patients with knee pain are indicated with red dots (Figure 2, lower row). Statistical differences between groups were widespread on the graph in the alpha band between 200 and 800 ms and in the beta band between 200 and 1,000 ms following movement initiation. These differences indicate an increase in alpha and beta power during movement execution.

### 3.3. Event related spectral perturbation in low back pain participants

**Figure 3:**
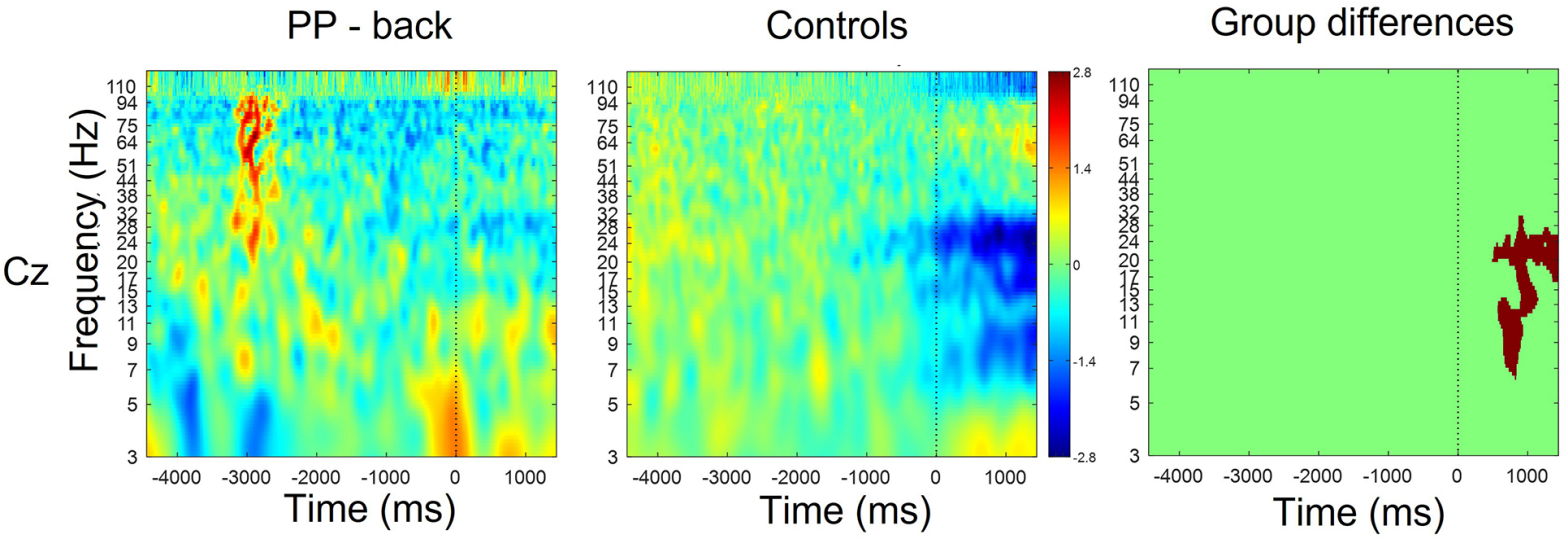
Time-frequency maps for controls and participants with low back pain over Cz. Movement onset is indicated by the vertical line (time 0). Regions of statistical differences (*p* < 0.05) are reported on the right column. Statistical differences were observed during the movement execution indicating an increase in oscillatory activity in the alpha and beta band in the participants with pain compared to healthy controls. No differences were observed in the resting phase between movements, that is until 2 s prior to movement initiation.

**Figure 4:**
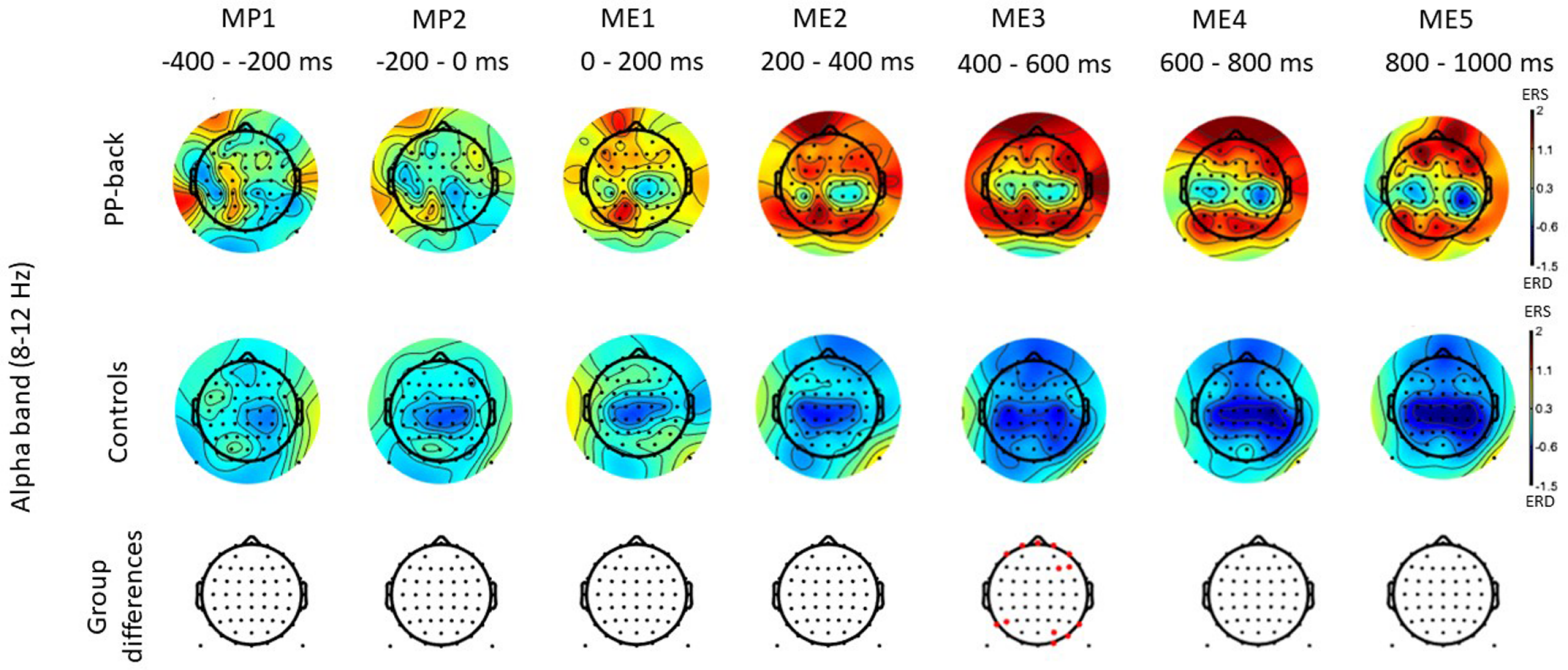
Scalp maps for the alpha and beta bands in time windows of 200 ms duration related to movement preparation (MP) and execution (ME) in participants with pain (PP) in the low back joint and healthy controls. The channels with statistical differences (*p* < 0.05) between controls and participants with pain are indicated in the lower row with red dots.

Figure 3 shows the time-frequency maps for control and patients with low back pain for Cz, as this electrode location is of primary somatotopic relevance for the leg area (12). Regions of statistical differences (*p* < 0.05) were observed in the Cz channel during movement execution (Figure 3, right column). No differences were observed during the resting phase that is until 2 s prior to movement initiation. The region of statistical difference indicates an increase in power in the alpha and beta bands. However, scalp maps do not show consistent differences in the alpha and beta bands between controls and PP. Figure 4 shows scalp maps for the alpha in time windows of 200 ms duration related to movement preparation and execution. In the figure, the channels with statistical differences (*p* < 0.05) between controls and patients with knee pain are indicated with red dots. Statistical differences are seen between groups in the alpha band related to only a few channel locations between 400 and 600 ms following movement initiation. No statistical differences were observed in the beta band.

## 4. Discussion

The current study evaluated differences in brain activity evoked during movement in participants with chronic knee or low back pain compared to healthy controls. The results indicate that brain activity was similar between pain participants and healthy controls in the resting phase between movements. During movement execution, however, participants with pain showed higher event related synchronization (ERS) in the alpha and beta bands. The region of statistical difference was larger in participants with knee pain.

Previous studies have implemented experimental pain models to investigate alterations in brain activity due to pain during resting conditions. For instance, studies using induction of deep-tissue pain through hypertonic saline injections showed an increase in beta power and a decrease in alpha power (28) compared to the condition involving no-pain. Research using tonic heat stimuli also showed a suppression of alpha power during tonic pain (29). In patient populations, no differences in brain activity have been observed between participants with central neuropathic pain and healthy volunteers during resting conditions (12). Paraplegic participants with central neuropathic pain, however showed an increased event related desynchronization in alpha, beta and theta band during imaginary movements (12). It is likely, though, that musculoskeletal pain would produce different cortical adaptations than those due to neuropathic pain. It is also likely that the movement execution may allow to reveal cortical adaptations not observable during resting or imaginary movements. Differences in brain activity between resting and movement states have been shown in patients suffering from tennis elbow pain (19). Using a model of pain where nerve growth factor is injected to the muscle of interest, induced long lasting pain (more than one week) and resulted in a decrease of the peak alpha frequency only during movement but not during the resting condition. This suggests that ongoing nociception might be necessary to show brain adaptations (30).

In the current study, no significant differences between participants with pain and healthy controls were found during the resting state between consecutive movements. During movement, however, participants with pain showed an increased ERS most consistently in the alpha and beta bands. The location of such adaptations seemed to be widespread and were consistently identified at Cz as expected due to the involvement of the sensory motor cortex in movement initiation and execution. Since beta activity has been associated with scanning mechanism related to perceptual and cognitive functions, the higher ERS in participants with pain, especially with knee pain, may be related to an increase in vigilance due to the occurrence of pain (25, 31). Alpha activity, instead, has been shown to possibly represent a consistent marker of sensory vs. internal processing (32). Alpha power has been documented to be a reliable and consistent measure (32), stable both in healthy individuals and in clinical population (33–35). The increase in desynchronization in the alpha band observed in this study in healthy participants from around movement initiation and proceeding into movement execution, is comparable with reports in previous literature. Alpha and beta band power decreases during movement initiation and execution and increases at the end of movement execution probably due to the termination of the movement (21). The desynchronization observed in healthy participants during movement initiation and execution could therefore indicate cortical activation necessary for motor planning. Other research has also described alpha blocking (decrease in alpha activity) in response to experimental stimuli as reflective of attentive stimulus processing, indicating states of increased vigilance or engagement with the external world (22, 32). On the contrary, alpha increases during tasks has been associated to internal cognitive processing, such as the use of working memory (36), and sensory suppression (37). The higher ERS observed in the current study in participants with pain could therefore be due to higher cognitive processing, possibly due to the attempt of suppressing the pain.

The higher ERS observed in pain participants compared to controls was similar between participants with knee pain and low back pain, though the region of statistical difference was larger in participants with knee pain. Since the movement is, as shown, a crucial element to induce nociceptive inputs and reveal difference in brain activity, it is possible that the region of differences was larger in participants with knee pain as the knee extension movement might have been easier to isolate.

One limitation of this study is the small sample size and the limited characterization of the pain participants (32). Results from previous studies have shown an effect size between 0.7 and 1 (12, 38). Given two groups with an equal number of subjects in each group, power calculations using Gpower suggest that a total of 12–24 subjects are needed to reach a power of 90% and a level of significance of 5% for an effect size of 1 and 0.7 between groups, respectively. Thus, the results of this short report will need to be confirmed with a larger sample size. All participants suffered from chronic pain, however, information about of frequency of pain might be necessary to evaluate whether the observed brain activity adaptations could be applied to other patients’ samples. However, it should be noted that both types of pain presented with similar changes as quantified by EEG as compared to healthy and pain free control participants. This is an important insight into how pain located at the periphery may be integrated at the level of the brain. Future studies need to further investigate if these alterations in brain activation patterns are similar across musculoskeletal pain sites and across stages of this type of pain (acute to chronic). This will provide knowledge on where the nociceptive feedback generated at the periphery is integrated within the nervous system to allow appropriate neurofeedback modalities to be developed.

The changes observed during movement execution in this short report study thus provide new knowledge contributing to our understanding of the neurophysiological changes associated with chronic pain that could possibly represent biomarkers of cortical plasticity. Moreover, modulating these neurophysiological biomarkers might be beneficial as pain treatment. The analgesic effect of altering brain oscillatory activity has previously been shown using meditation (39). Also promising results following neuromodulator approaches such as neurofeedback, have been shown in cases of, for instance, chronic neuropathic pain, fibromyalgia and migraine (11, 38, 40).

### 4.1. Conclusion

The current short report investigates the changes in cortical activities during movement preparation and execution in participants with chronic knee and low back pain, confirming that specific changes are associated to movement execution and possibly related to the ongoing nociceptive input produced by the movement. These results provide insights that could be useful in understanding the neurophysiological changes related to chronic pain, identify markers associated with the occurrence of pain and the development of new modulatory treatment approaches.

## Data Availability

The raw data supporting the conclusions of this article will be made available by the authors, without undue reservation.
